# Multimorbidity adjusted years lost to disability rates calculated through Monte-Carlo simulation in Korea

**DOI:** 10.4178/epih.e2022090

**Published:** 2022-10-17

**Authors:** Yoonhee Shin, Eun Jeong Choi, Bomi Park, Hye Ah Lee, Eun-Kyung Lee, Hyesook Park

**Affiliations:** 1 Department of Preventive Medicine, Ewha Womans University College of Medicine, Seoul, Korea; 2 College of Nursing, Ewha Womans University, Seoul, Korea; 3 Department of Preventive Medicine, Chung-Ang University College of Medicine, Seoul, Korea; 4 Clinical Trial Center, Mokdong Hospital, Ewha Womans University, Seoul, Korea; 5 Department of Statistics, Ewha Womans University, Seoul, Korea; 6 Graduate Program in System Health Science and Engineering, Ewha Womans University, Seoul, Korea

**Keywords:** Multimorbidity, Years lived with disability, Monte Carlo method, Korea

## Abstract

**OBJECTIVES:**

To efficiently utilize limited health and medical resources, it is necessary to accurately measure the level of health, which requires estimating the multimorbidity-corrected burden of disease.

**METHODS:**

This study used 2015 and 2016 data from the National Health Insurance Service, and employed the list of diseases defined in a Korean study of the burden of disease, the criteria for prevalence, and the “cause–sequelae–health state” disease system. When calculating the years lost to disability (YLD), multimorbidity was corrected using Monte-Carlo simulation.

**RESULTS:**

Correcting for multimorbidity changed YLD at all ages in Korea by −1.2% (95% confidence interval [CI], −24.1 to 3.6) in males and −12.4% (95% CI, −23.0 to 0.3) in females in 2015, and by −10.8% (95% CI, −24.1 to 4.6) in males and −11.1% (95% CI, −22.8 to 1.7) in females in 2016. The YLD rate for non-communicable diseases in males decreased more than that of other disease groups in both years, by −11.8% (95% CI, −19.5 to 3.6) and −11.5% (95% CI, −19.3 to −3.0), respectively. The overall YLD rate changed by −1.3% in the 5-year to 9-year age group, and the magnitude of this change remained similar until the 10–19-year age group, gradually decreased after 20 years of age, and steeply increased to more than 10% in those aged 60 and older.

**CONCLUSIONS:**

Calculations of YLD should adjust for multimorbidity, as the disease burden can otherwise be overestimated for the elderly, who tend to exhibit a high prevalence of multimorbidity.

## GRAPHICAL ABSTRACT

[Fig f2-epih-44-e2022090]

## INTRODUCTION

Health indicators are often measured to implement health care policies as efficiently as possible while utilizing limited health care resources [1]. Although death and chronic disease indicators have traditionally been used to measure health, these cross-sectional indicators have limitations as indicators of health levels between countries [2,3]. To solve these problems, the World Health Organization (WHO) calculated the burden of disease in the Global Burden of Disease (GBD) study with a single health level metric that included both disease and death due to risk factors [4,5].

Disability-adjusted life years (DALY), an indicator of disease burden used in the GBD, are calculated as the difference between total years of life lost and years lost to disability (YLD). The DALY quantitatively measures the health gap between ideal and actual health conditions, indicating the degree to which specific causes contribute to death or disease. It can also be used to indicate the health status of a group or to compare the health levels of 2 groups. The DALY is used to identify the social burden of diseases and to implement public health policies designed to reduce this social burden with limited health care resources. It can also be used to evaluate and analyze the implementation of health and medical policies. Therefore, the importance of the DALY continues to be emphasized in the field of public health.

The prevalence of multimorbidity, defined as having 2 or more diseases at the same time, has been increasing worldwide due to the increase in chronic diseases and population aging [6,7]. In Korea, the prevalence of multimorbidity in the overall population was 34.8% in 2014 [8]. The prevalence of multimorbidity in South Asia increased from 4.5% in 2000 to 83% in 2015 [9]. Several studies have consistently reported the risks associated with multimorbidity and reductions in quality of life due to multimorbidity [10,11], suggesting that multiple diseases cause more personal and social problems than do single diseases, and that these problems rapidly lead to increased social costs [12,13]. Considering the increasing prevalence of multimorbidity and its large impact on health and quality of life, it is necessary to measure the burden of disease more accurately to facilitate the efficient use of limited resources. To achieve this, corrections should be made for multimorbidity when measuring the burden of disease.

The first GBD study calculated the disease burden without considering multimorbidity. Beginning in 2010, disease burden was calculated by assuming independent prevalence rates for each disease and then considering multimorbidity [4]. In Korea, research on the disease burden has been conducted steadily since the late 1990s. Initially, the disease burden of Koreans was calculated without considering multimorbidity, but this began to change in 2017. The scope of corrected diseases has gradually expanded, but remains limited [14]. Therefore, it is meaningful to evaluate differences in disease burden according to multimorbidity adjustments.

This study sought to estimate the effectiveness of adjusting for the prevalence of co-occurring diseases in calculating the burden of disease by calculating YLD rates considering the prevalence of multimorbidity and comparing the results with the YLD rates calculated without doing so. In addition, we examined the statistical accuracy of this method of correcting for multimorbidity by calculating the YLD rates and comparing the values with 2 independent sources.

## MATERIALS AND METHODS

The prevalence-based YLD rates were calculated as the prevalence of each health sequela multiplied by its disability weight (DW).

$$\begin{array}{c} \text{Prevalence-based YLD rate}=\\ \text{Prevalence of disease }{\text{sequela}}^{★}\text{DW of the sequela} \end{array}$$

In this study, the list of diseases, the criteria for measuring prevalence, and the “cause–sequelae–health state” disease system defined in the Korean Burden of Disease Study [15] were used to define the prevalence status and calculate prevalence based YLD rates. However, the assumption of mutual exclusivity among health sequelae (i.e., the assumption that the health sequelae of a disease exist independently of one another, and that several health sequelae may exist at the same time for one person) was used to supplement the cause–sequelae–health state disease system defined in the Korean Burden of Disease Study.

This study analyzed data from sex (male, female) aged over 5 years in Korea. The YLD rates were calculated by age, sex, and year, using 9 age groups: 5–9 years, 10–19 years, 20–29 years, 30–39 years, 40–49 years, 50–59 years, 60–69 years, 70–79 years, and 80 years or older. Those aged 0–4 years were excluded from the analysis due to the low disease prevalence rates.

The prevalence of causes by sex–age group was calculated based on 2015 and 2016 data from the National Health Insurance Service, and the prevalence of health sequelae was estimated using the prevalence distribution of health sequelae by cause identified in a systematic review. In addition, for acute cases or chronic diseases with short prevalence periods, such as sudden infant death syndrome, the prevalence period was corrected to estimate the prevalence. The prevalence-based YLD rates were calculated for 260 causes of diseases. In 2015, but not in 2016, claims data covering traditional medicine were included. Multiplicative methods were applied to the health-state DW values calculated by Ock et al. [16] to estimate DWs for health sequelae.

The multimorbidity-adjusted YLD rate for each disease can be calculated as the sum of the YLDs of the disease sequela. To calculate YLD rates corrected for co-occurring diseases, Monte-Carlo simulation was performed during the last stage of the YLD rate calculation. This creates a virtual population with various combinations of sequelae by assuming the exposure to each sequela based on probability [17]. In other words, to calculate YLD rates corrected for multimorbidity, assuming that the simulant was exposed to each sequela independently, a Monte-Carlo simulation was performed based on the prevalence of each sequela to create 40,000 simulants for multimorbidity for each age–sex–year combination. The DW of each simulant was calculated by applying a multiplicative approach to the DW of each health state according to the health sequelae of the simulant.

$$Simulant\;DW_{l}=1-\prod_{k=1}^{j}\left(1-DW_{k}\right)$$

*DW_k_*= the *DW* for *k* disease sequela that simulant *I* has acquired

The DW of each simulant was allocated to each disease sequela constituting the simulant using the formula below.

$${ADW}_{lk}=\frac{{DW}_{k}}{\sum_{k=i}^{k=j}{DW}_{k}}*Simulant\;{DW}_{l}$$

*ADW_lk_*=attributable *DW* of phase sequela *k* belonging to simulant *lDWl*=*DW* for the combination of sequela experienced by simulant *I*

The YLD rates corrected for multimorbidity were calculated by averaging the DW obtained for 40,000 simulants.

$$YLD\;{Rate}_{k}=\frac{\sum_{l=1}^{n}ADW_{lk}}{n}$$

The same method used in the GBD 2016 study allowed 40,000 simulants to be created in 1 simulation, and 1,000 repetitions for each sex–age group were performed to obtain the uncertainty [5,18]. The YLD rate for a sequela obtained by adjusting for multimorbidity was compared with the rate calculated without adjustment for multimorbidity.

The Monte-Carlo simulation was performed using R version 3.6.0 (R Foundation, Vienna, Austria), and the diseases were categorized into 5 groups according to previous research on disease burden measurement and prediction in Koreans: (1) non-communicable diseases (NCDs); (2) communicable diseases (CDs); (3) injuries; (4) maternal, neonatal, and nutritional conditions (MNNs); and (5) mental disorders (MDs).

### Ethics statement

This study was approved by the Korea University Institutional Review Board (KU-IRB-18-EX-51-A-1). The requirement for informed consent was waived.

## RESULTS

Adjusting for multimorbidity changed the YLD rates at all ages in Korea by −11.2% (95% CI, −24.1 to 0.0) in males and −12.4% (95% CI, −23.0 to −0.3) in females in 2015, and by −10.8% (95% CI, −24.1 to 0.0) in males and −11.1% (95% CI, −22.8 to 0.0) in females in 2016 (Table 1).

When examined by disease group, adjusting for multimorbidity changed the YLD rate for NCDs in males the most in both years (−11.8 and −11.5%, respectively). In 2015, the next largest change was observed for MDs (−10.9%), followed by CDs (−10.0%), MNNs (−9.6%), and injuries (−9.3%). In 2016, the corresponding order was MDs (−10.4%), MNNs (−9.0%), injuries (−9.0%), and CDs (−8.9%). For females, multimorbidity adjustment decreased the YLD rate of NCDs significantly, by −12.6% in 2015 and −11.6% in 2016. However, among females, the effect of multimorbidity adjustment showed the largest annual variation in MNNs, with the largest drop (−13.8%) in 2015 and the smallest drop (−3.2%) in 2016. Thus, for females, adjusting for multimorbidity reduced the YLD rates in the following descending order by disease group: MNNs (−13.8%), NCDs (−12.6%), MDs (−12.2%), injuries (−11.9%), and CDs (−8.6%) in 2015, versus NCDs (−11.6%), MDs (−10.9%), injuries (−10.9%), CDs (−7.5%), and MNNs (−3.2%) in 2016 (Table 1).

By age group, adjusting for multimorbidity decreased the overall YLD rate by −1.5% in those 5–9 years of age, while a slightly larger decrease (by −2 to −6%) was observed in those ≥20 years of age, and significant decreases (with magnitudes exceeding 10%) were found in subjects aged ≥ 60 years (Table 1). Older ages were associated with greater decreases in YLD rates after multimorbidity adjustment, with the greatest decrease found in males over 80 years (−18.0%) in 2015 and in females 70 years and older (−17.9%) in 2015. In 2016, the decline was the greatest among those over 80 in both sexes (Table 1). As age increased, the YLD rates tended to show larger decreases for each disease group (Table 2). However, there was no significant change in the YLD rank among diseases across ages. For some diseases, the YLD ranking varied by more than 3 steps, but there was no meaningful change in the overall YLD rankings, even in older people who showed large drops in YLD rates (Table 3).

**Figure 1:**
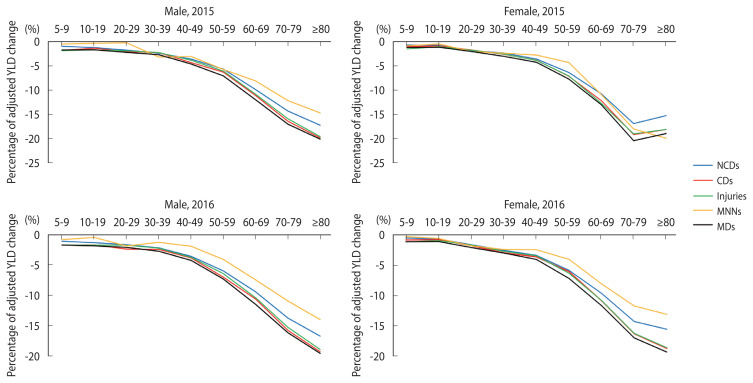
Change in adjusted YLD by age group in 2015 and 2016 (per 1,000 person). YLD, years lost to disability; NCD, non-communicable diseases; CDs, communicable diseases; MNNs, maternal, neonatal, and nutritional conditions; MDs, mental disorders.

In an analysis of the changes in YLD rates after multimorbidity correction for age group in 2015 and 2016, both males and females showed the smallest changes in MNNs, while MDs presented the largest changes (Figure 1).

## DISCUSSION

After adjustment for multimorbidity, the overall YLD rates in 2015 and 2016 decreased, with changes of about −10% in both males and females compared to the pre-calibration values. The magnitude of the change increased with age, beginning gradually after the age of 20, and increasing steeply in those over 60, reaching more than −10.0%.

The YLD rates adjusted for multimorbidity decreased in both males and females across all age groups, confirming that the failure to adjust for multimorbidity when calculating disease burden could lead to an overestimation of YLD rates. Given the longer life expectancy and accelerated aging of the population, the prevalence of multimorbidity is increasing, which will increase the burden of multimorbidity among the elderly. Therefore, if YLD rates are calculated without considering multimorbidity in older people with a high prevalence of multiple diseases, the disease burden may be overestimated. Thus, it is important to adjust for multimorbidity when calculating YLD rates. This finding confirms a report by Hilderink et al. [19], who studied changes in YLD rates based on corrections for multimorbidity in 25 diseases. However, because this study used data from 2015 and 2016, the results may be different from those calculated based on more recent data.

A review of the decrease in the YLD rate by disease group confirmed that males and females of all ages exhibited decreased YLD rates for MDs when multimorbidity was considered (Figure 1). An analysis of the changes in the ranking of YLD rates before and after adjustment for multimorbidity revealed that the ranks of some illnesses in this category rose or fell, but without any particularly noticeable changes in the ranking (Table 3). Therefore, multimorbidity adjustment did not affect the relative YLD rates of most diseases within a disease group. Indeed, there was no noticeable change in the ranking of YLD rates in any disease category other than mental illness. Therefore, further research is needed on the development of appropriate correction methods for each disease group because the change in YLD after correction for multimorbidity may vary among disease types.

In the 2016 analysis, the YLD rates by sex (male, female), disease type, and age were slightly lower than those in 2015, which included claims involving traditional medicine. Further research using applied data is needed to obtain stable annual trends. In addition, there may be cases where the prevalence of particular diseases is low due to the age distribution or where YLD rates depend on the characteristics of diseases such as maternal and infant conditions. Furthermore, for those under 20 years of age, Monte-Carlo simulation results may be misleading due to the low prevalence of multimorbidity; this contingency must also be considered when calculating YLD rates (Table 1 and Supplementary Materials 1 and 2).

In the GBD study and the Netherlands study [20], the prevalence and DWs of comorbid diseases were calculated under the assumption that each disease was carried independently, and the YLD rate was simulated to compensate for multimorbidity. In this study, as in the GBD study, the YLD rate adjusted for multimorbidity was calculated under the assumption of independent diseases. However, as noted in the Australian Disease Burden Study, it should be considered that the prevalence or DW of severe diseases in individuals with multiple diseases of different severities affects the post-calibration outcomes [21]. It would be ideal if the prevalence and DWs of multiple diseases could be measured directly when calculating the burden of disease considering multimorbidity, but it is practically difficult to calculate the prevalence and DWs for combinations of multimorbidity in all cases.

Mathers et al. [22] reported that it is more accurate to assume that multiple diseases are dependent on one another than to assume that each disease is independent when evaluating YLD rates in health-adjusted life expectancy calculations. However, it is difficult to determine the probability of multimorbidity by considering the sequence of occurrence of all possible combinations of diseases. This suggests that the disease burden can be overestimated if the YLD rate is calculated without considering multimorbidity. Therefore, the disease burden should be calculated with consideration of co-occurring diseases, and a realistic methodology that assumes dependence among diseases is needed. In Korea, the disease burden corrected in consideration of several diseases is calculated, and the scope of these diseases is expanding [14]. For domestic disease burden research in the future, a method for calculating the disease burden with correction for co-occurring diseases should be developed in line with the GBD research methodology.

In the present study, we used a Monte-Carlo simulation method to correct for multimorbidity by combining all diseases in a virtual simulation. This method has the advantages of preventing overestimation of the disease burden when calculating the YLD rate without considering multimorbidity and of enabling an evaluation of disease burden with adjustment for multimorbidity, without the need to account for temporal changes in the proportions of major multimorbid diseases. However, the use of Monte-Carlo simulation to adjust for all diseases can result in an overestimation of the YLD rate. Therefore, a follow-up study is needed to assess the suitability of Monte-Carlo simulations based on data generated using a method that compensates for multiple diseases.

## Figures and Tables

**Figure f2-epih-44-e2022090:**
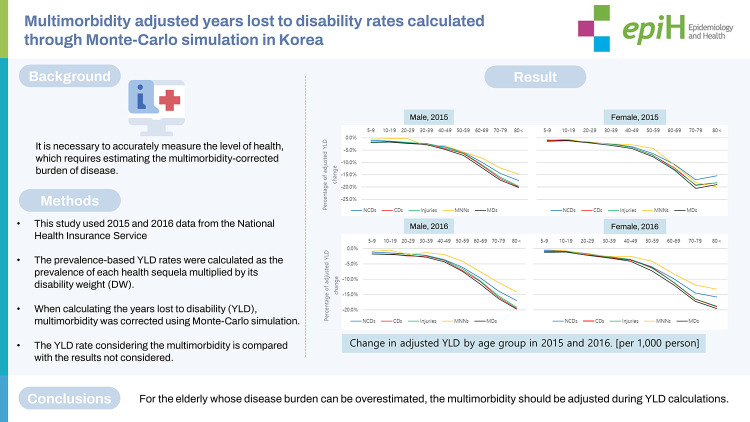


**Table 1.** Multimorbidity-adjusted YLD rates through Monte-Carlo simulations in 2015-2016 (per 1,000 population)

Varoables			Male				Female			
			Prevalence	Unadjusted YLD rates	Adjusted YLD rates (95% CI)	% Change (95% CI)	Prevalence	Unadjusted YLD rates	Adjusted YLD rates (95% CI)	% Change (95% CI)
2015			5,758.1	2,285.6	2029.8 (1,735.7-2,368.0)	-11.2 (-24.1-3.6)	6,122.9	2,468.6	2,161.6 (1,899.9-2,460.7)	-12.4 (-23.0-0.3)
	By disease									
		NCDs	4,054.4	1,476.6	1,302.3 (1,189.0, 1,423.7)	-11.8 (-19.5, -3.6)	4,224.9	1,524.9	1,332.6 (1,223, 1,448.2)	-12.6 (-19.8, -5.0)
		CDs	88.3	41.8	37.6 (30.7, 44.9)	-10.0 (-26.6, 7.5)	84.0	38.3	35.0 (28.6, 42.8)	-8.6 (-25.3, 11.9)
		Injuries	900.0	393.3	356.8 (229.8, 515.9)	-9.3 (-41.6, 31.2)	717.5	325.0	286.4 (196.2, 401.0)	-11.9 (-39.6, 23.4)
		MNNs	25.7	4.7	4.3 (2.7, 6.3)	-9.6 (-41.7, 33.7)	309.8	139.9	120.6 (112.2, 130.5)	-13.8 (-19.8, -6.7)
		MDs	689.7	369.1	328.8 (283.6, 377.1)	-10.9 (-23.2, 2.2)	786.6	440.6	387.0 (339.8, 438.1)	-12.2 (-22.9, -0.6)
	By age									
		5-9	104.8	34.6	34.1 (27.1, 42.8)	-1.5 (-21.5, 23.8)	76.0	23.8	23.6 (18.3, 30.4)	-1.0 (-23.0, 27.7)
		10-19	164.4	67.3	66.2 (45.7, 91.5)	-1.6 (-32.1, 36.0)	100.2	40.0	39.6 (25.8, 57.2)	-1.0 (-35.4, 43.0)
		20-29	180.6	81.1	79.5 (54.2, 109.7)	-2.0 (-33.2, 35.3)	179.7	79.1	77.5 (56.4, 103.4)	-2.0 (-28.7, 30.7)
		30-39	233.9	102.2	99.7 (72.2, 133.8)	-2.4 (-29.4, 30.9)	258.0	108.6	105.7 (81.8, 134.6)	-2.6 (-24.6, 24.0)
		40-49	376.3	162.6	156.2 (121.4, 197.8)	-3.9 (-25.4, 21.6)	385.8	161.1	154.9 (124.8, 190.2)	-3.8 (-22.5, 18.1)
		50-59	636.6	262.4	246.6 (207.4, 291.6)	-6.0 (-21.0, 11.1)	710.9	290.3	270.5 (231.3, 314.3)	-6.8 (-20.3, 8.2)
		60-69	1,179.9	459.4	411.8 (361.1, 468.6)	-10.4 (-21.4, 2.0)	1306.7	509.7	451.1 (403.3, 504.3)	-11.5 (-20.9, -1.1)
		70-79	1,774.1	685.4	582.7 (523.0, 647.6)	-15.0 (-23.7, -5.5)	2107.1	862.7	708.2 (652.3, 768.7)	-17.9 (-24.4, -10.9)
		≥80	1,107.5	430.5	353.0 (323.6, 384.5)	-18.0 (-24.8, -10.7)	998.4	393.4	330.6 (305.8, 357.7)	-16.0 (-22.3, -9.1)
2016			5,623.4	2237.0	1,996.0 (1,700.0, 2339.4)	-10.8 (-24.1, 4.6)	5,627.1	2,256.0	2,005.7 (1,742.6, 2,294.5)	-11.1 (-22.8, 1.7)
	By disease									
		NCDs	3,887.7	1,418.3	1,255.8 (1,144.9, 1,375.1)	-11.5 (-19.3, -3.0)	3,872.7	1,404.2	1,241.7 (1,136.6, 1,355.5)	-11.6 (-19.1, -3.5)
		CDs	90.8	42.8	39.0 (31.8, 46.8)	-8.9 (-25.6, 9.4)	88.5	40.2	37.2 (30.1, 44.9)	-7.5 (-25.0, 11.8)
		Injuries	953.0	414.1	376.9 (244.6, 542.3)	-9.0 (-40.9, 30.9)	717.8	320.2	285.2 (192.2, 400.6)	-10.9 (-40.0, 25.1)
		MNNs	26.0	4.6	4.2 (2.8, 6.2)	-9.0 (-40.6, 34.0)	165.3	47.1	45.6 (38.6, 53.3)	-3.2 (-18.0, 13.2)
		MDs	666.0	357.2	320.1 (275.9, 368.9)	-10.4 (-22.7, 3.3)	782.8	444.3	396.0 (345.1, 440.3)	-10.9 (-22.3, -0.9)
	By age									
		5-9	101.6	34.2	33.6 (26.5, 42.6)	-1.5 (-22.5, 24.6)	61.6	20.7	20.5 (15.2, 27.4)	-0.9 (-26.3, 32.4)
		10-19	176.4	73.0	71.7 (50.3, 97.8)	-1.8 (-31.0, 34.0)	108.5	43.6	43.2 (28.7, 62.1)	-1.0 (-34.1, 42.3)
		20-29	175.7	78.9	77.4 (53.4, 107.1)	-1.9 (-32.3, 35.7)	172.5	76.8	75.3 (54.3, 101.5)	-1.9 (-29.3, 32.3)
		30-39	234.0	102.0	99.5 (71.8, 133.7)	-2.4 (-29.6, 31.1)	282.0	119.9	116.5 (91.6, 146.8)	-2.8 (-23.6, 22.4)
		40-49	368.1	158.7	152.6 (118.3, 194.2)	-3.8 (-25.4, 22.4)	365.7	152.8	147.2 (117.8, 182.0)	-3.7 (-22.9, 19.1)
		50-59	663.5	273.8	256.3 (212.7, 307.6)	-6.4 (-22.3, 12.3)	647.0	269.2	252.1 (213.2, 296.6)	-6.3 (-20.8, 10.2)
		60-69	1,129.7	440.5	396.7 (346.6, 453.0)	-9.9 (-21.3, 2.8)	1,178.9	458.1	410.8 (360.9, 461.4)	-10.3 (-21.2, 0.7)
		70-79	1,706.7	657.5	562.9 (504.6, 626.6)	-14.4 (-23.3, 4.7)	1,802.7	702.5	596.3 (542.7, 654.5)	-15.1 (-22.8, -6.8)
		≥80	1,067.7	418.4	345.1 (315.7, 376.8)	-17.5 (-24.5, -9.9)	1,008.2	412.5	343.8 (318.2, 362.3)	-16.6 (-22.9, -12.2)

YLD, years lost to disability; NCD, non-communicable diseases; CDs, communicable diseases; MNNs, maternal, neonatal, and nutritional conditions; MDs, mental disorders.

**Table 2.** Prevalence rate and multimorbidity-adjusted YLD rates by age and disease group in 2015-2016 (per 1,000 population)

Age (yr)	Disease	2015								2016							
		Male				Female				Male				Female			
		Pre valence	Un adjusted YLD rates	Adjusted YLD rates	Change in YLD rates (%)	Pre valence	Un adjusted YLD rates	Adjusted YLD rates	Change in YLD rates (%)	Pre valence	Un adjusted YLD rates	Adjusted YLD rates	Change in YLD rates (%)	Pre valence	Un adjusted YLD rates	Adjusted YLD rates	Change in YLD rates (%)
5-9	NCDs	54.6	13.4	13.2	-1.0	46.7	11.5	11.4	-0.7	52.2	13.0	12.8	-1.1	30.0	7.1	7.1	-0.6
	CDs	11.2	4.8	4.7	-1.8	11.3	4.9	4.8	-1.1	12.8	5.7	5.6	-1.8	13.0	5.7	5.7	-0.9
	Injuries	18.0	7.8	7.7	-1.9	9.9	4.3	4.2	-1.6	16.7	7.2	7.0	-1.7	11.1	4.9	4.8	-1.3
	MNNs	1.2	0.3	0.3	-0.5	1.0	0.2	0.2	-0.9	1.1	0.3	0.3	-0.9	1.0	0.2	0.2	-0.4
	MDs	19.8	8.3	8.1	-1.8	46.7	2.9	2.9	-1.2	18.8	8.1	8.0	-1.8	30.0	2.7	2.7	-1.2
10-19	NCDs	39.5	11.8	11.6	-1.2	34.3	10.4	10.3	-0.8	42.2	12.4	12.3	-1.4	35.1	10.5	10.4	-0.8
	CDs	8.3	3.6	3.6	-1.4	8.8	3.8	3.8	-0.9	13.5	6.3	6.2	-1.7	10.8	4.8	4.8	-0.9
	Injuries	70.8	29.7	29.2	-1.8	24.7	10.6	10.5	-1.1	74.2	31.2	30.7	-1.8	29.1	12.2	12.1	-1.0
	MNNs	1.0	0.2	0.2	-0.4	6.8	1.2	1.2	-0.5	1.0	0.2	0.2	-0.5	6.9	1.2	1.2	-0.7
	MDs	44.7	22.0	21.7	-1.7	25.6	14.0	13.8	-1.2	45.5	22.9	22.4	-1.9	26.5	14.9	14.8	-1.2
20-29	NCDs	37.4	14.3	14.0	-1.8	45.6	16.5	16.2	-1.9	35.9	13.3	13.0	-1.7	42.1	14.7	14.5	-1.7
	CDs	3.8	1.8	1.8	-2.1	6.2	2.9	2.8	-1.8	3.7	1.7	1.7	-2.4	6.2	2.8	2.8	-1.9
	Injuries	95.8	39.9	39.2	-1.9	51.7	22.1	21.7	-1.8	90.9	37.8	37.2	-1.8	50.7	21.5	21.1	-1.7
	MNNs	0.6	0.1	0.1	-0.3	30.4	10.9	10.6	-2.1	0.6	0.1	0.1	-1.9	25.5	9.0	8.9	-1.9
	MDs	42.9	24.9	24.4	-2.2	45.8	26.8	26.2	-2.1	44.5	26.0	25.5	-2.2	47.9	28.7	28.1	-2.2
30-39	NCDs	85.4	33.0	32.2	-2.4	100.0	36.3	35.4	-2.4	88.9	34.2	33.4	-2.2	95.7	34.3	33.4	-2.7
	CDs	5.6	2.6	2.5	-2.3	7.5	3.4	3.3	-2.6	5.6	2.5	2.5	-2.5	9.3	4.1	4.0	-3.0
	Injuries	97.6	40.8	39.9	-2.3	45.9	19.6	19.1	-2.6	93.9	39.0	38.1	-2.3	47.4	20.3	19.7	-2.8
	MNNs	0.8	0.2	0.2	-3.2	49.8	17.5	17.1	-2.4	0.8	0.2	0.2	-1.3	67.2	24.3	23.7	-2.5
	MDs	44.5	25.6	24.9	-2.7	54.8	31.8	30.8	-3.1	44.9	26.1	25.4	-2.8	62.5	36.8	35.7	-3.1
40-49	NCDs	209.7	81.5	78.6	-3.6	201.5	76.2	73.4	-3.6	200.3	77.3	74.5	-3.6	183.5	68.8	66.4	-3.5
	CDs	6.2	2.9	2.8	-4.4	7.6	3.4	3.3	-3.9	6.1	2.9	2.7	-3.9	7.7	3.5	3.3	-3.7
	Injuries	96.3	40.8	39.3	-3.9	63.0	27.0	26.0	-3.9	98.4	41.5	39.9	-3.8	63.8	27.2	26.2	-3.6
	MNNs	1.0	0.2	0.2	-3.1	33.0	7.0	6.8	-2.8	1.0	0.2	0.2	-2.0	32.7	6.9	6.7	-2.6
	MDs	63.2	37.1	35.4	-4.6	80.7	47.4	45.4	-4.3	62.2	36.8	35.2	-4.3	78.0	46.5	44.6	-4.2
50-59	NCDs	460.0	173.7	163.7	-5.7	482.8	175.6	164.3	-6.4	441.4	166.2	156.1	-6.1	437.6	159.2	149.7	-6.0
	CDs	8.2	3.9	3.7	-6.3	8.8	4.0	3.7	-7.2	7.7	3.7	3.4	-7.1	8.5	3.8	3.6	-6.2
	Injuries	84.4	37.1	34.8	-6.1	97.0	44.0	40.8	-7.1	132.6	57.1	53.4	-6.5	99.3	42.6	39.9	-6.4
	MNNs	1.9	0.4	0.3	-5.8	9.1	1.6	1.5	-4.3	1.8	0.3	0.3	-4.2	8.8	1.4	1.4	-4.1
	MDs	82.2	47.4	44.0	-7.1	113.3	65.2	60.1	-7.8	80.0	46.5	43.1	-7.4	92.8	62.2	57.6	-7.3
60-69	NCDs	912.8	331.7	298.7	-10.0	988.1	348.8	311.0	-10.8	866.7	315.0	285.0	-9.5	897.7	316.9	285.9	-9.8
	CDs	12.3	5.9	5.3	-11.2	11.6	5.3	4.6	-12.2	11.5	5.4	4.9	-10.7	11.0	5.0	4.4	-11.0
	Injuries	141.4	61.6	54.9	-10.9	136.0	63.0	55.0	-12.8	142.3	61.7	55.2	-10.5	108.5	47.7	42.5	-11.0
	MNNs	4.1	0.8	0.7	-8.2	6.1	1.2	1.1	-11.0	3.9	0.7	0.6	-7.5	5.4	1.0	0.9	-8.2
	MDs	109.3	59.4	52.3	-12.1	165.0	91.4	79.4	-13.1	105.4	57.7	51.0	-11.6	156.3	87.5	77.2	-11.8
70-79	NCDs	1,381.6	497.5	425.9	-14.4	1,495.1	534.3	443.6	-17.0	1,327.1	478.7	412.4	-13.9	1,376.9	494.2	422.9	-14.4
	CDs	18.6	9.1	7.6	-16.5	14.5	6.9	5.6	-19.3	17.1	8.3	6.9	-15.9	13.5	6.3	5.3	-16.5
	Injuries	187.8	85.2	71.6	-16.0	183.1	84.0	68.0	-19.1	194.2	87.5	74.1	-15.4	190.0	87.3	73.0	-16.4
	MNNs	8.1	1.4	1.2	-12.2	166.0	98.9	81.0	-18.1	8.5	1.5	1.3	-11.0	10.2	1.8	1.6	-11.9
	MDs	178.0	92.2	76.4	-17.1	248.5	138.5	110.1	-20.5	159.9	81.5	68.2	-16.3	212.0	112.9	93.5	-17.2
≥80	NCDs	873.4	319.7	264.3	-17.3	830.8	315.2	266.8	-15.3	832.9	308.2	256.2	-16.9	774.2	298.4	251.4	-15.7
	CDs	14.1	7.1	5.7	-19.9	7.8	3.8	3.1	-18.2	12.8	6.4	5.1	-19.4	8.4	4.1	3.3	-18.9
	Injuries	107.9	50.4	40.5	-19.7	106.4	50.4	41.2	-18.2	109.9	51.0	41.3	-19.1	117.7	56.6	46.0	-18.8
	MNNs	7.0	1.3	1.1	-14.8	7.7	1.4	1.1	-20.0	7.2	1.3	1.1	-14.1	7.6	1.3	1.2	-13.3
	MDs	105.1	52.0	41.5	-20.2	45.8	22.7	18.4	-19.1	104.9	51.5	41.4	-19.7	100.3	52.0	41.9	-19.5

YLD, years lost to disability; NCD, non-communicable diseases; CDs, communicable diseases; MNNs, maternal, neonatal, and nutritional conditions; MDs, mental disorders.

**Table 3.** Changes of 3 steps or more in rankings of adjusted YLD rates by age and sex (male, female) in 2015 and 2016

Age (yr)		Sex	Ranking change	Disease	Cause
2015					
	5-9	Male	▼4	CDs	Respiratory syncytial virus pneumonia
		Female	▲3	MDs	Drug-Opioid disorder
	10-19	Male	▲3	MDs	Drug-Opioid disorder
		Female	▼3	CDs	H influenza, type B pneumonia
	20-29	Male	▲5	NCDs	Other urinary organ cancer
	60-69	Female	▼3	MNNs	Protein-energy malnutrition
	70-79	Female	▲6	NCDs	Chronic kidney disease due to diabetes mellitus
			▼5	MDs	Drug-Other drug use disorders
	≥80	Female	▼4	NCDs	Migraine
2016					
	5-9	Male	▼3	MDs	Drug-Amphetamine use disorder
	10-19	Male	▲3	MDs	Drug-Cannabis use disorder
			▼3	NCDs	Pancreatic cancer
	20-29	Female	▲3	NCDs	Multiple myeloma
			▼3	MDs	Drug-Amphetamine use disorder
	40-49	Female	▼3	Injuries	Fire, heat, and hot substances
	70-79	Male	▼3	MDs	Bipolar affective disorder
		Female	▼3	NCDs	Pancreatic cancer

YLD, years lost to disability; NCD, non-communicable diseases; CDs, communicable diseases; MNNs, maternal, neonatal, and nutritional conditions; MDs, mental disorders.
