# Supplementary material for: Multimorbidity adjusted years lost to disability rates calculated through Monte-Carlo simulation in Korea
Source: Epidemiol Health. 2022 Oct 17;44:e2022090. doi: 10.4178/epih.e2022090 (PMC10089703; doi:10.4178/epih.e2022090)
Supplement: Supplementary file 1 [file epih-44-e2022090-Supplementary-1.docx]

**Supplemental Table 1.** Multimorbidity-adjusted YLD rates calculated using Monte Carlo simulation in 2015–2016 (per 1000 population).

|  | Total (Male and Female) | | |  |
| --- | --- | --- | --- | --- |
|  | Unadjusted YLD rates | Adjusted  YLD rates (95% CI) | Percentage change  (95% CI) | |
| 2015 | 2382.6 | 2095.8 (1818-2414.4) | -12.0% (-31.1-0) * | |
| By disease |  |  |  | |
| NCDs | 1502.9 | 1317.5 (1206.1-1436) | -12.3% (-24.6--4.7) | |
| CDs | 39.2 | 36.3 (29.6-43.9) | -7.4% (-32.4-0) * | |
| Injuries | 360.6 | 321.6 (213-458.3) | -10.8% (-69.3-0) * | |
| MNNs | 77.1 | 62.5 (57.6-68.5) | -18.9% (-33.9--12.6) | |
| MDs | 402.7 | 357.9 (311.7-407.7) | -11.1% (-29.2-0) * | |
| By age |  |  |  | |
| 5–9 | 29.4 | 29 (22.9-36.8) | -1.4% (-28.4-0) * | |
| 10–19 | 54.2 | 53.5 (36.2-75.1) | -1.3% (-49.7-0) * | |
| 20–29 | 80.2 | 78.5 (55.2-106.7) | -2.1% (-45.3-0) * | |
| 30–39 | 105.3 | 102.7 (76.9-134.2) | -2.5% (-36.9-0) * | |
| 40–49 | 161.9 | 155.6 (123.1-194.1) | -3.9% (-31.5-0) * | |
| 50–59 | 276.4 | 258.5 (219.3-302.9) | -6.5% (-26.0-0) * | |
| 60–69 | 485.5 | 432 (382.8-486.9) | -11.0% (-26.8-0) * | |
| 70–79 | 785.0 | 654.6 (597.1-716.9) | -16.6% (-31.5--9.5) | |
| 80< | 404.7 | 337.4 (311.2-365.8) | -16.6% (-30.0--10.6) | |
| 2016 | 2246.5 | 2000.9 (1721.3-2316.9) | -10.9% (-23.4-0) * | |
| By disease |  |  |  | |
| NCDs | 1411.2 | 1248.7 (1140.7-1365.3) | -11.5% (-19.2--3.3) | |
| CDs | 41.5 | 38.1 (31-45.9) | -8.2% (-25.3-0) * | |
| Injuries | 367.1 | 330.9 (218.3-471.3) | -9.8% (-40.5-0) * | |
| MNNs | 25.9 | 25 (20.7-29.8) | -3.7% (-20.0-0) * | |
| MDs | 400.8 | 358.2 (310.6-404.7) | -10.7% (-22.5-0) * | |
| By age |  |  |  | |
| 5–9 | 27.6 | 27.3 (21-35.2) | -1.3% (-23.8-0) * | |
| 10–19 | 58.9 | 58 (40-80.7) | -1.5% (-32.1-0) * | |
| 20–29 | 77.9 | 76.4 (53.8-104.5) | -1.9% (-30.9-0) * | |
| 30–39 | 110.8 | 107.9 (81.5-140.1) | -2.6% (-26.4-0) * | |
| 40–49 | 155.8 | 149.9 (118.1-188.2) | -3.8% (-24.2-0) * | |
| 50–59 | 271.5 | 254.2 (213-302.1) | -6.4% (-21.6-0) * | |
| 60–69 | 449.5 | 404 (353.9-457.3) | -10.1% (-21.3-0) * | |
| 70–79 | 683.1 | 581.9 (526.4-642.6) | -14.8% (-22.9--5.9) | |
| 80< | 414.3 | 344.2 (317.4-366.7) | -16.9% (-23.4--11.5) | |

NCDs (non-communicable diseases), CDs (communicable diseases), MNNs (maternal, neonatal, and nutritional conditions), MDs (mental disorders).

* The 95% confidence interval upper limit of the percentage change in the reduction can only be negative, so it should be interpreted as 0.
