# Supplementary material for: Multimorbidity adjusted years lost to disability rates calculated through Monte-Carlo simulation in Korea
Source: Epidemiol Health. 2022 Oct 17;44:e2022090. doi: 10.4178/epih.e2022090 (PMC10089703; doi:10.4178/epih.e2022090)
Supplement: Supplementary file 2 [file epih-44-e2022090-Supplementary-2.docx]

**Supplemental Table 2**. Multimorbidity-adjusted YLD rates according to age and disease group in 2015–2016 (per 1000 population).

| Age  Group | Disease  Group | Total (Male and Female) | | | | | |
| --- | --- | --- | --- | --- | --- | --- | --- |
|  |  | 2015 | | | 2016 | | |
|  |  | Unadjusted YLD rates | Adjusted YLD rates | Change in YLD rates | Unadjusted YLD rates | Adjusted YLD rates | Change in YLD rates |
| 5–9 | NCDs | 12.5 | 12.4 | -0.9% | 10.1 | 10.0 | -1.0% |
|  | CDs | 4.9 | 4.8 | -1.5% | 5.7 | 5.6 | -1.4% |
|  | Injuries | 6.1 | 6.0 | -1.8% | 6.1 | 6.0 | -1.6% |
|  | MNNs | 0.2 | 0.2 | -0.7% | 0.2 | 0.2 | -0.6% |
|  | MDs | 5.7 | 5.6 | -1.6% | 5.5 | 5.4 | -1.6% |
| 10–19 | NCDs | 11.1 | 11.0 | -1.1% | 11.5 | 11.4 | -1.1% |
|  | CDs | 3.7 | 3.7 | -1.1% | 5.6 | 5.5 | -1.4% |
|  | Injuries | 20.6 | 20.2 | -1.6% | 22.1 | 21.8 | -1.6% |
|  | MNNs | 0.7 | 0.7 | -0.5% | 0.6 | 0.6 | -0.7% |
|  | MDs | 18.2 | 17.9 | -1.5% | 19.1 | 18.8 | -1.6% |
| 20–29 | NCDs | 15.3 | 15.0 | -1.8% | 13.9 | 13.7 | -1.7% |
|  | CDs | 2.3 | 2.3 | -1.9% | 2.2 | 2.2 | -2.1% |
|  | Injuries | 31.5 | 30.9 | -1.9% | 30.1 | 29.5 | -1.8% |
|  | MNNs | 5.2 | 5.1 | -2.1% | 4.4 | 4.3 | -1.9% |
|  | MDs | 25.8 | 25.3 | -2.2% | 27.3 | 26.7 | -2.2% |
| 30–39 | NCDs | 34.6 | 33.8 | -2.4% | 34.3 | 33.4 | -2.4% |
|  | CDs | 3.0 | 2.9 | -2.5% | 3.3 | 3.2 | -2.8% |
|  | Injuries | 30.4 | 29.7 | -2.4% | 29.9 | 29.1 | -2.5% |
|  | MNNs | 8.7 | 8.4 | -2.4% | 12.0 | 11.7 | -2.5% |
|  | MDs | 28.7 | 27.8 | -2.9% | 31.3 | 30.4 | -3.0% |
| 40–49 | NCDs | 78.9 | 76.0 | -3.6% | 73.1 | 70.5 | -3.6% |
|  | CDs | 3.2 | 3.0 | -4.1% | 3.2 | 3.0 | -3.8% |
|  | Injuries | 34.0 | 32.7 | -3.9% | 34.5 | 33.2 | -3.7% |
|  | MNNs | 3.6 | 3.5 | -2.8% | 3.5 | 3.4 | -2.5% |
|  | MDs | 42.2 | 40.3 | -4.5% | 41.6 | 39.8 | -4.2% |
| 50–59 | NCDs | 174.7 | 164.0 | -6.1% | 162.7 | 152.9 | -6.0% |
|  | CDs | 4.0 | 3.7 | -6.8% | 3.7 | 3.5 | -6.6% |
|  | Injuries | 40.6 | 37.9 | -6.7% | 49.9 | 46.7 | -6.5% |
|  | MNNs | 1.0 | 0.9 | -4.6% | 0.9 | 0.8 | -4.1% |
|  | MDs | 56.2 | 52.0 | -7.5% | 54.3 | 50.3 | -7.3% |
| 60–69 | NCDs | 340.6 | 305.1 | -10.4% | 316.0 | 285.5 | -9.7% |
|  | CDs | 5.6 | 4.9 | -11.7% | 5.2 | 4.6 | -10.9% |
|  | Injuries | 62.4 | 55.0 | -11.9% | 54.5 | 48.6 | -10.7% |
|  | MNNs | 1.0 | 0.9 | -9.9% | 0.8 | 0.8 | -7.9% |
|  | MDs | 75.9 | 66.2 | -12.7% | 73.1 | 64.5 | -11.7% |
| 70–79 | NCDs | 518.6 | 436.2 | -15.9% | 487.6 | 418.4 | -14.2% |
|  | CDs | 7.8 | 6.4 | -17.9% | 7.2 | 6.0 | -16.2% |
|  | Injuries | 84.6 | 69.6 | -17.7% | 87.5 | 73.5 | -16.0% |
|  | MNNs | 55.5 | 45.5 | -18.1% | 1.6 | 1.5 | -11.5% |
|  | MDs | 118.4 | 95.5 | -19.4% | 99.4 | 82.6 | -16.9% |
| 80+ | NCDs | 316.6 | 266.1 | -16.0% | 301.4 | 252.9 | -16.1% |
|  | CDs | 4.8 | 3.9 | -19.0% | 4.8 | 3.9 | -19.1% |
|  | Injuries | 50.4 | 41.0 | -18.6% | 54.9 | 44.5 | -18.9% |
|  | MNNs | 1.3 | 1.1 | -18.5% | 1.3 | 1.1 | -13.5% |
|  | MDs | 31.6 | 25.4 | -19.6% | 51.9 | 41.7 | -19.6% |
